# Prediction of cardiac autonomic dysfunction using heart rate response to deep breathing test among type 2 diabetes mellitus

**DOI:** 10.1186/s12902-025-01939-8

**Published:** 2025-04-25

**Authors:** Sohini Raje, G. Arun Maiya, Padmakumar R, Mukund A. Prabhu, Krishnananda Nayak, K.N. Shivashankara, B.A. Shastry, Megha Nataraj, Shreemathi S. Mayya

**Affiliations:** 1 Centre for Podiatry and Diabetic Foot Care and Research, Department of Physiotherapy, Manipal College of Health Professions, Manipal Academy of Higher Education (MAHE), Manipal, 576104 Karnataka India; 2 Department of Cardiology, Kasturba Medical College- Manipal, Manipal Academy of Higher Education (MAHE), Manipal, 576104 Karnataka India; 3 Department of Cardiovascular Technology, Manipal College of Health Professions, Manipal Academy of Higher Education (MAHE), Manipal, 576104 Karnataka India; 4 Department of Medicine, Kasturba Medical College- Manipal, Manipal Academy of Higher Education (MAHE), Manipal, 576104 Karnataka India; 5 Department of Cardiovascular & Respiratory Physiotherapy, MGM College of Physiotherapy, Navi Mumbai, 400705 Maharashtra India; 6 MGM Hospital & Research Centre, CBD Belapur, Navi Mumbai, 400614 Maharashtra India; 7 Department of Data Science, Prasanna School of Public Health, Manipal Academy of Higher Education (MAHE), Manipal, 576104 Karnataka India

**Keywords:** Cardiovascular tests, Autonomic function, Cross-sectional study, Diabetic neuropathy, Complication

## Abstract

**Background:**

Cardiac autonomic neuropathy (CAN) is an underdiagnosed complication of type 2 diabetes mellitus (T2DM) and a predictor of mortality and cardiovascular morbidity. Hence, CAN screening is essential. The objective of the study was to examine whether cardiac autonomic dysfunction can be predicted using the heart rate response to deep breathing test of cardiac autonomic reflex tests (CARTs) among type 2 diabetes mellitus.

**Methods:**

The study was a cross-sectional study of T2DM individuals between 40 and 65 years. Each participant underwent a heart rate (HR) response to deep breathing test (CARTs) as per standard guidelines. ANOVA F-test was used to check the difference between the CAN severity and the heart rate response to deep breathing parameters. A post-hoc (Tukey’s) test was used to check which groups showed the difference.

**Results:**

Eighty-four participants were screened, of which forty-one were included in the present study. The mean age of the participants was 58.8 ± 4.0 years. The Fisher’s test showed a statistically significant difference between groups for the average deep breathing difference (F(3,27) = 16.09, *p* < 0.001) and the respiratory sinus arrhythmia index (F(3,27) = 7.35, *p* < 0.001).

**Conclusion:**

HR response to deep breathing can be used as a preliminary tool to screen CAN in T2DM to differentiate between normal and the other stages of CAN, which can then be followed by the gold standard tests. Further studies are required to establish HR response to deep breathing as a singular tool using regression analysis.

**Clinical Trials Registration:**

The study was registered prospectively in the Clinical Trials Registry- India (CTRI/2023/11/060077) on 21st November 2023.

## Background

Diabetes mellitus (DM) is a public health burden of the 21st century. The prevalence of DM is about 21.4% in India, with 90% of individuals with diabetes mellitus having type 2 diabetes mellitus (T2DM) [1]. Out of the myriad of end-organ complications, one of them is cardiac autonomic neuropathy (CAN). CAN is defined as the “impairment of the autonomic nervous system in the setting of diabetes after ruling out all other causes” [2]. CAN is recognized as an indicator of mortality, morbidity, and subclinical cardiovascular dysfunction and events [3, 4]. The prevalence of CAN is reported to be between 31 and 73% in T2DM [5]. The Toronto Consensus considers the following reasons to screen for CAN in individuals with DM: (1) To diagnose and stage CAN; (2) For cardiovascular risk stratification and risk of other microvascular complications of DM; (3) To adjust glycemic control based on the CAN stage. Thus, it is essential to screen for CAN among T2DM individuals [6].

The screening and diagnosis of cardiac autonomic neuropathy can be performed by one of the following methods: cardiac autonomic reflex tests (CARTs), heart rate variability (HRV) analysis, and baroreflex sensitivity (BRS). CARTs and HRV are non-invasive measures, while baroreflex sensitivity is an invasive method for assessing cardiac autonomic function [7]. CARTs are a series of non-invasive bedside tests that utilize heart rate and blood pressure by changing positions and altering breathing patterns to test parasympathetic and sympathetic systems. They are considered the gold standard [8]. The following tests are included in the CARTs- heart rate (HR) response with deep breathing, HR response with standing, Valsalva maneuver, postural blood pressure change, and sustained handgrip test. While the tests have good sensitivity and specificity, individual tests have shortcomings. For example, obtaining the Valsalva ratio from the Valsalva maneuver is essentially effort-dependent [9], or the postural hypotension test is usually positive in the severe stage of CAN.

The maximum variation in HRV is seen with deep breathing [10]. In a retrospective analysis, HR response to deep breathing test was described as a single test for CAN screening based on accuracy, followed by Valsalva maneuver [11]. Another analysis reported the lying to standing test and Valsalva maneuver [12, 13]. A study by Abdelwanis et al. utilized a machine learning algorithm to predict CAN by using a two-stage approach and used inflammatory markers and Ewing’s tests to make predictions based on each test [14]. A previous meta-analysis also reported the association of diabetic peripheral neuropathy (DPN) with CAN. They found about 58.9% of individuals with CAN also had DPN, with significant heterogeneity between studies [15]. However, there is no clear consensus about utilizing a single test for preliminary screening of CAN in T2DM.

In a clinical setting, performing all tests to screen for CAN in T2DM may not be possible. Hence, there is a need to explore a single test for screening for CAN in T2DM individuals [6]. The objective of the present study was to examine whether heart rate response to deep breathing test can predict cardiac autonomic neuropathy in individuals with type 2 diabetes mellitus.

## Materials and methods

The study was a cross-sectional study done in a tertiary university teaching hospital. The study was carried out per the Declaration of Helsinki. The study was approved by the Institutional Ethics Committee (IEC:322/2023) and registered in the Clinical Trials Registry- India (CTRI/2023/11/060077). The study is part of a larger ongoing trial.

### Inclusion criteria

Individuals were screened if they were physician-diagnosed type 2 diabetes mellitus for more than one year, aged between 40 and 65 years, of either gender.

### Exclusion criteria

The exclusion criteria were type 1 diabetes mellitus, gestational diabetes mellitus, or any other genetic forms: the presence of any underlying conditions such as recent myocardial infarction or coronary artery bypass graft, rheumatic heart disease, abdominal surgery in the past three months, chronic kidney disease, acute kidney injury, diabetic retinopathy, pulmonary condition, malignancy or neuromuscular disorder.

### Sample size

The G Power software version 3.1.9.7 was used to calculate the sample size. A sample size of 41 was considered necessary with an effect size of 0.50 and power 0.80 (80%) and 0.05 α alpha level. The sampling method was convenience sampling for the present study.

### Procedure

If the participant satisfies the inclusion criteria, written informed consent is obtained from the participant. The participant underwent cardiac autonomic reflex testing (CARTs) (Ewing’s Tests) using the KODYS Cardiac Autonomic Neuropathy Analyser (KODYSCAN) (CAN-126; Key no. 2956 3665 4589) to obtain the respiratory sinus arrhythmia (RSA) index, average deep breathing difference (avg. DBD) and the expiration to inspiratory (E: I) ratio. We performed the five Ewing’s tests (HR response to deep breathing, lying to standing test, Valsalva maneuver, orthostatic hypotension test, blood pressure change with sustained handgrip). However, the HR response to deep breathing test will be considered for the analysis. The tests were performed between 8 am and 9 am in a dimly lit room at a constant temperature of 22 °C to 24 °C. The tests were performed with participants fasting (for at least eight hours) to avoid consuming caffeine at least 2 h before the test, avoiding strenuous physical activity (24 h prior), smoking, and wearing loose, comfortable clothing.

#### Heart rate response to deep breathing test

The test was conducted as per the guidelines given by Ewing et al. [8]. The participant was asked to be in the supine lying position and was guided to perform deep breathing for one minute with six seconds for inspiration and six seconds for expiration. Three parameters were obtained: average deep breathing difference (Avg. DBD), respiratory sinus arrhythmia (RSA) index, and expiration: inspiration (E: I) ratio. Avg. DBD is the “difference in heart rate is calculated by measuring the difference between the average of the three highest heart rates and the average of the three lowest heart rates over the six breathing cycles”. E: I ratio is “the ratio between the average of the three longest RR intervals during expiration and the average of the three shortest RR intervals during inspiration” [6].

### Statistical analysis

Data was analyzed using Jamovi 2.3.21 software. The characteristics of the participants were analyzed using descriptive statistics. The normality of the data was checked using the Shapiro-Wilk test. In addition, the visual assessment for the normality was performed using histograms. If the criteria of normality were satisfied, mean and standard deviation were used to report the data. Median and interquartile range was reported if the data (or outcome) was not normally distributed. The ANOVA F-Test (one-way ANOVA for grouped means) was used to check the difference between the groups for the CAN severity and the HR response to deep breathing variables. CAN severity was considered in four stages per Ewing’s criteria (Ewing et al., 1985). The Fisher’s test was applied, and descriptive statistics were used to report the mean and standard deviation of the HR response to deep breathing variables according to the stage. A post-hoc test (Tukey’s test) was used to check which groups showed the difference if the *p*-value between the groups was statistically significant (< 0.05).

## Results

84 participants were screened, of which 41 were included in the present study. The mean age of the participants was 58.8 ± 4.0 years. 26 participants were males (*n* = 26; 63.4%). The body mass index was 25.7 ± 3.5 kg/m^2^. Maximum individuals had a duration between 6 and 10 years (16/41), followed by 10 to 15 years (12/41), more than 15 years (6/41), and 1 to 5 years (7/41). Most participants were on a diet and oral hypoglycemic agents (about 40%). The participants had the following comorbidities– hypertension (*n* = 12; 29.2%), dyslipidemia (*n* = 3; 7.3%), hypothyroidism (*n* = 7; 17.0%), and musculoskeletal condition (*n* = 2; 4.8%). The participants were on the following medications– angiotensin receptor blockers (*n* = 6; 14.6%), angiotensin-converting enzyme inhibitors (*n* = 2; 4.8%), beta-blockers (*n* = 4; 9.7%), statins (*n* = 3; 7.3%), calcium channel blockers (*n* = 6; 14.6%) and thyroxine supplements (*n* = 8; 19.5%). Characteristics of the participants are mentioned in Table 1.

**Table 1:** Characteristics of the participants

	Mean ± SD/median(IQR)
Age (years)	58.6 ± 5.2
Height (cm)	168.0 ± 9.7
Weight (kg)	73.2 ± 13.7
BMI (kg/m^2^)	25.7 ± 3.5
HbA1C (%)	7.5 ± 1.5
FBS (mg/dl)	137.0 ± 40.0
PPBS (mg/dl)	176.0 (88.5)
**HR response to deep breathing**	
Avg. DBD (bpm) RSA Index E: I ratio	11.7 ± 6.9 0.2 ± 0.1 1.1 ± 0.07

Abbreviation- BMI- Body mass index; HbA1C- glycated hemoglobin; FBS- fasting blood sugar; PPBS- postprandial blood sugar; HR- heart rate; Avg. DBD- average deep breathing difference; RSA Index- respiratory sinus arrhythmia index

### Relationship between the CAN severity and the HR response to deep breathing parameters

The group descriptives are shown in Table 2. The Fisher’s test showed a statistically significant difference between groups for the average deep breathing difference (F(3,27) = 16.09, *p* < 0.001) and the respiratory sinus arrhythmia index (F(3,27) = 7.35, *p* < 0.001). There was no statistically significant difference between the groups for the E: I ratio (F(3,27) = 1.39, *p* = 0.13).

**Table 2:** Group descriptives

	CAN Severity	*n*	Mean ± SD
Avg. DBD (bpm)	Normal	8	20.6 ± 7.1
	Early	16	12.7 ± 4.7
	Definite	7	6.2 ± 3.9
	Severe	10	6.7 ± 2.7
RSA Index	Normal	8	0.4 ± 0.2
	Early	16	0.2 ± 0.1
	Definite	7	0.1 ± 0.04
	Severe	10	0.1 ± 0.07
E: I ratio	Normal	8	1.1 ± 0.1
	Early	16	1.1 ± 0.1
	Definite	7	1.1 ± 0.02
	Severe	10	1.1 ± 0.1

Abbreviations- Avg. DBD- average deep breathing difference; RSA Index- respiratory sinus arrhythmia index; E: I ratio- expiration: inspiration ratio; n- number of participants

The post-hoc test (Tukey’s test) was applied to check which groups showed the difference in the average deep breathing difference and the respiratory sinus arrhythmia index. A statistically significant difference was observed between the normal stage of CAN and the three stages (early, definite, severe) for average deep breathing difference and the respiratory sinus arrhythmia index. Similarly, a statistically significant difference was observed between the early and definite, and early and severe stages. Tables 3 and 4 show the difference between the stages for average deep breathing difference and the respiratory sinus arrhythmia index.

**Table 3:** Tukeys post-hoc test for the average deep breathing difference

		Early	Definite	Severe
Normal	MD	7.86	14.35	13.88
	*p*-value	0.003	< 0.001*	< 0.001*
Early	MD	-	6.49	6.01
	*p*-value	-	0.024	0.018
Definite	MD	-	-	-0.471
	*p*-value	-	-	0.997
Severe	MD	-	-	-
	*p*-value	-	-	-

Abbreviations- MD- mean difference; *- statistically significant *p*-value

**Table 4:** Tukeys post-hoc test for the RSA index

		Early	Definite	Severe
Normal	MD	0.18	0.29	0.29
	*p*-value	0.025	0.002	< 0.001
Early	MD	-	0.10	0.10
	*p*-value	-	0.381	0.300
Definite	MD	-	-	-0.002
	*p*-value	-	-	1.00
Severe	MD	-	-	-
	*p*-value	-	-	-

Abbreviations- MD- mean difference

## Discussion

The present study showed a statistically significant difference between normal CAN and the other stages of CAN when average deep breathing difference and the respiratory sinus arrhythmia index were used to measure the severity of CAN in individuals with T2DM. Thus, the results show that the HR response to deep breathing test has the capability to differentiate between normal CAN and abnormal (early, definite, or severe) CAN.

Previous studies hypothesized resting heart rate as a predictor for cardiac autonomic dysfunction and future cardiovascular events in the general population and individuals with T2DM [16, 17]. One of the limitations of using resting heart rate is that it is influenced by the frequency of standing and HRV [18]. In the case of the HR response to deep breathing test, the parameters obtained are not influenced by resting heart rate unless it is more than 100 beats per minute [6].

The study by Bellavere et al. reported a high specificity of 88% for using a combination of the deep breathing test and the lying to standing test [13]. Pafili et al. also reported the 30:15 ratio (obtained from the lying to standing test) to have a good potential to predict CAN in T2DM [12]. In the present study, the HR response to deep breathing test could differentiate between the normal and abnormal CAN. The lying-to-standing test can be added as an adjunct to the deep breathing test to perform the preliminary screening for CAN. This initial screening can then be followed by the gold standard Ewing’s test to confirm the diagnosis of CAN [13]. One of the advantages of considering the HR response to deep breathing test is that methodological differences for the test can be minimized to a greater degree, which may not be possible for the other tests in the CARTs [6]. In the present study, the mean values of the deep breathing test parameters were lower (Table 1). Lower values of these parameters were reported to be associated with higher coronary calcium scores and, thereby, the risk of developing coronary atherosclerosis [19] (Engstrom et al., 2021).

Exercise training is an essential aspect of lifestyle management of type 2 diabetes mellitus, which helps in glycemic control, improving HbA1C, improving muscle mass, and cardiovascular risk factors [20]. Exercise training has been shown to improve cardiac autonomic function in type 2 diabetes mellitus [21–23]. Physical therapists often administer deep breathing, segmental breathing exercises, and respiratory muscle training to most individuals to improve their pulmonary function, quality of life, and functional capacity in various systemic conditions [24–26]. Although there are few studies on the effect of respiratory muscle training and breathing exercises on improvements in cardiac autonomic, these are shown to have a positive impact [27]. Future studies can examine the effect of segmental breathing exercises on the cardiac autonomic reflex test variables in T2DM.

CAN is a significant complication and is an independent predictor of all-cause and cardiovascular mortality in T2DM [28]. It is an independent risk factor for arrhythmia, silent ischemia, and myocardial dysfunction [29]. The pathogenesis of CAN is complex and multifactorial. The presence of hyperglycemia, genetic predisposition, inflammation, age-related neuronal death, and obstructive sleep apnea in individuals with type 2 diabetes mellitus make them more susceptible to the development of CAN [30, 31]. The symptoms of CAN (e.g., orthostatic hypotension, exercise intolerance) appear in the severe stage, with pre-existing systemic conditions. Thus, there is a need and scope for a preliminary test (such as HR response to deep breathing) for screening in a clinical setting, which can be followed by the utilization of other methods of diagnosis, which can aid in strengthening the pharmacological and non-pharmacological management strategies.

One of the strengths of the present study is that the study population resembles the real world, where a co-existence of type 2 diabetes mellitus occurs with other comorbidities. Also, the present study demonstrated the practical utility of cardiac autonomic reflex tests in a clinical setting. Our study had a few limitations- we did not control for the medications. However, participants were asked not to consume medications that alter blood pressure and heart rate before the testing. Also, the minute variations in the breathing pattern cannot be controlled; participants were given standard instructions to avoid significant variations between individuals. Potential bias in the study may have occurred due to the inclusion criteria (selection bias). Participants with other comorbidities, such as hypertension and hypothyroidism, could not be excluded as these often coexist in individuals with T2DM, and therefore, practically, it is difficult to have individuals with only T2DM. However, the comorbidities were controlled, and participants were on pharmacological management for the same. Further studies with larger sample sizes can perform logistic regression analysis to check the association between the HR response to deep breathing tests and the cardiac autonomic neuropathy severity.

## Conclusion

The present study showed the HR response to deep breathing test as a potential screening tool to differentiate between normal and abnormal cardiac autonomic neuropathy in type 2 diabetes mellitus. The cardiac autonomic reflex tests are not routinely performed in the clinical setting. Individuals with type 2 diabetes mellitus are at risk of falls [32, 33] (one of the causes being postural hypotension). Hence, performing all tests may not be feasible. Considering a comprehensive assessment that is performed in all individuals with type 2 diabetes mellitus, the HR response to deep breathing test can be used routinely in a heavily engaged clinical setting to screen for CAN. However, further studies with a larger sample size to facilitate regression analysis are required to establish the same. The study falls within the United Nations Sustainable Development Goal − 3 (Good Health and Wellbeing).

## Data Availability

The data generated and analyzed during the current study are not publicly available but are available from the corresponding author on reasonable request.

## References

[1] Sun H, Saeedi P, Karuranga S, Pinkepank M, Ogurtsova K, Duncan BB, et al. IDF diabetes atlas: global, regional and country-level diabetes prevalence estimates for 2021 and projections for 2045. Diabetes Res Clin Pract. 2022;183:109119. 34879977 10.1016/j.diabres.2021.109119 PMC11057359

[2] Tesfaye S, Boulton AJM, Dyck PJ, Freeman R, Horowitz M, Kempler P, et al. Diabetic neuropathies: update on definitions, diagnostic criteria, Estimation of severity, and treatments. Diabetes Care. 2010;33:2285. 20876709 10.2337/dc10-1303 PMC2945176

[3] Rolim LC, de Souza JST, Dib SA. Tests for early diagnosis of cardiovascular autonomic neuropathy: critical analysis and relevance. Front Endocrinol (Lausanne). 2013;4 NOV:2–5. 24273533 10.3389/fendo.2013.00173 PMC3822331

[4] Jakubiak GK, Chwalba A, Basek A, Cieślar G, Pawlas N. Glycated hemoglobin and cardiovascular disease in patients without diabetes. J Clin Med 2025. 2024;14:14:53. 10.3390/jcm14010053 PMC11721913 39797136

[5] Williams S, Raheim SA, Khan MI, Rubab U, Kanagala P, Zhao SS, et al. Cardiac autonomic neuropathy in type 1 and 2 diabetes: epidemiology, pathophysiology, and management. Clin Ther. 2022;44:1394–416. 36272822 10.1016/j.clinthera.2022.09.002

[6] Spallone V, Bellavere F, Scionti L, Maule S, Quadri R, Bax G, et al. Recommendations for the use of cardiovascular tests in diagnosing diabetic autonomic neuropathy. Nutr Metabolism Cardiovasc Dis. 2011;21:69–78. 10.1016/j.numecd.2010.07.005 21247746

[7] Pop-Busui R. Cardiac autonomic neuropathy in diabetes: A clinical perspective. Diabetes Care. 2010;33:434–41. 20103559 10.2337/dc09-1294 PMC2809298

[8] Ewing D, Martyn C, Young R, Clarke B. The value of cardiovascular autonomic. Diabetes Care. 1985;8:491–8. 4053936 10.2337/diacare.8.5.491

[9] Ewing DJ, Campbell IW, Clarke BF. Assessment of cardiovascular effects in diabetic autonomic neuropathy and prognostic implications. Ann Intern Med. 1980;92(2):308–11. 7356219 10.7326/0003-4819-92-2-308

[10] Sridhar B, Haleagrahara N, Bhat R, Kulur AB, Avabratha S, Adhikary P. Increase in the heart rate variability with deep breathing in diabetic patients after 12-month exercise training. Tohoku J Exp Med. 2010;220:107–13. 20139661 10.1620/tjem.220.107

[11] Stranieri A, Abawajy J, Kelarev A, Huda S, Chowdhury M, Jelinek HF. An approach for ewing test selection to support the clinical assessment of cardiac autonomic neuropathy. Artif Intell Med. 2013;58:185–93. 23768975 10.1016/j.artmed.2013.04.007

[12] Pafili K, Trypsianis G, Papazoglou D, Maltezos E, Papanas N. Simplified diagnosis of cardiovascular autonomic neuropathy in type 2 diabetes using Ewing’s battery. Rev Diabet Stud. 2015;12:213–9. 26676669 10.1900/RDS.2015.12.213 PMC5397991

[13] Bellavere F, Ragazzi E, Chilelli NC, Lapolla A, Bax G. Autonomic testing: which value for each cardiovascular test? An observational study. Acta Diabetol. 2019;56:39–43. 30159748 10.1007/s00592-018-1215-y

[14] Abdelwanis M, Moawad K, Mohammed S, Hummieda A, Syed S, Maalouf M et al. Sequential classification approach for enhancing the assessment of cardiac autonomic neuropathy. 2025. 10.1016/j.compbiomed.2025.109999 10.1016/j.compbiomed.2025.109999 40112561

[15] De Lima AV, Dykstra GM, Barbosa Da Rocha R, Magalhães AT, Kato Da Silva A, Saura Cardoso V. The association of diabetic peripheral neuropathy with cardiac autonomic neuropathy in individuals with diabetes mellitus: A systematic review. 2024. 10.1016/j.jdiacomp.2024.108802 10.1016/j.jdiacomp.2024.108802 38971002

[16] Tang ZH, Zheng F, Li Z, Zhou L. Association and Predictive Value Analysis for Resting Heart Rate and Diabetes Mellitus on Cardiovascular Autonomic Neuropathy in General Population. J Diabetes Res. 2014;2014. 10.1155/2014/215473 PMC3977100 24772443

[17] Koskela JK, Tahvanainen A, Tikkakoski AJ, Kangas P, Uitto M, Viik J, et al. Resting heart rate predicts cardiac autonomic modulation during passive head-up Tilt in subjects without cardiovascular diseases. Scandinavian Cardiovasc J. 2022;56:138–47. 10.1080/14017431.2022.2079713 35652524

[18] Alexander J, Sovakova M, Rena G. Factors affecting resting heart rate in free-living healthy humans. Digit Health. 2022;8. 10.1177/20552076221129075 PMC9549087 36225988

[19] Engström G, Hamrefors V, Fedorowski A, Persson A, Johansson ME, Ostenfeld E et al. Cardiovagal function measured by the deep breathing test: relationships with coronary atherosclerosis. J Am Heart Assoc. 2022;11. 10.1161/JAHA.121.024053 PMC9075454 35352566

[20] Joseph JJ, Deedwania P, Acharya T, Aguilar D, Bhatt DL, Chyun DA, et al. Comprehensive management of cardiovascular risk factors for adults with type 2 diabetes. A Scientific Statement from the American Heart Association; 2022. 10.1161/CIR.0000000000001040 35000404

[21] Picard M, Tauveron I, Magdasy S, Benichou T, Bagheri R, Ugbolue UC et al. Effect of exercise training on heart rate variability in type 2 diabetes mellitus patients: A systematic review and meta-analysis. PLoS ONE. 2021;16. 10.1371/journal.pone.0251863 PMC8128270 33999947

[22] Hamasaki H. The effect of exercise on cardiovascular autonomic nervous function in patients with diabetes: A systematic review. Healthc (Switzerland). 2023;11. 10.3390/healthcare11192668 PMC10572826 37830705

[23] Zaki S, Alam MF, Sharma S, El-Ashker S, Ahsan M, Nuhmani S. Impact of concurrent exercise training on cardiac autonomic modulation, metabolic profile, body composition, cardiorespiratory fitness, and quality of life in type 2 diabetes with cardiac autonomic neuropathy: A randomized controlled trial. J Clin Med. 2024;13:1–25. 10.3390/jcm13133910 PMC11242881 38999476

[24] Yang Y, Wei L, Wang S, Ke L, Zhao H, Mao J, et al. The effects of pursed lip breathing combined with diaphragmatic breathing on pulmonary function and exercise capacity in patients with COPD: a systematic review and meta-analysis. Physiother Theory Pract. 2022;38:847–57. 32808571 10.1080/09593985.2020.1805834

[25] Fan J, Chang Y, Cheng S, Liang B, Qu D. Effect of breathing exercises on patients with interstitial lung disease: A systematic review and meta-analysis. Qual Life Res. 2024. 10.1007/s11136-024-03679-z. 38907831 10.1007/s11136-024-03679-z

[26] Fabero-Garrido R, del Corral T, Plaza-Manzano G, Sanz-Ayan P, Izquierdo-García J, López-De-Uralde-Villanueva I. Effects of respiratory muscle training on exercise capacity, quality of life, and respiratory and pulmonary function in people with ischemic heart disease: systematic review and Meta-Analysis. Phys Ther. 2024;104. 10.1093/ptj/pzad164 38015997

[27] Shao R, Man ISC, Lee TMC. The effect of Slow-Paced breathing on cardiovascular and emotion functions: A Meta-Analysis and systematic review. Mindfulness (N Y). 2024;15:1–18.

[28] Ang L, Dillon B, Mizokami-Stout K, Pop-Busui R. Cardiovascular autonomic neuropathy: A silent killer with long reach. Auton Neurosci. 2020;225:102646. 32106052 10.1016/j.autneu.2020.102646

[29] Pop-Busui R, Boulton AJM, Feldman EL, Bril V, Freeman R, Malik RA, et al. Diabetic neuropathy: A position statement by the American diabetes association. Diabetes Care. 2017;40:136–54. 27999003 10.2337/dc16-2042 PMC6977405

[30] Fisher VL, Tahrani AA. Cardiac autonomic neuropathy in patients with diabetes mellitus: current perspectives. Diabetes Metabolic Syndrome Obes. 2017;10:419–34. 10.2147/DMSO.S129797 PMC5638575 29062239

[31] Agashe S, Petak S. Cardiac autonomic neuropathy in diabetes mellitus. Methodist Debakey Cardiovasc J. 2018;14:251–6. 30788010 10.14797/mdcj-14-4-251 PMC6369622

[32] Freire LB, Brasil-Neto JP, da Silva ML, Miranda MGC, de Mattos Cruz L, Martins WR, et al. Risk factors for falls in older adults with diabetes mellitus: systematic review and meta-analysis. BMC Geriatr. 2024;24:1–12. 38413865 10.1186/s12877-024-04668-0 PMC10900672

[33] Yang Y, Hu X, Zhang Q, Zou R. Diabetes mellitus and risk of falls in older adults: a systematic review and meta-analysis. Age Ageing. 2016;45:761–7. 27515679 10.1093/ageing/afw140

